# Assessing Psychological Harms on Parents and Primary Caregivers of Children Living with a Rare Disease: A Systematic Review of the Scope and Validity of Surveys Utilized

**DOI:** 10.1007/s10567-025-00533-7

**Published:** 2025-06-30

**Authors:** Lochlan J. Bull, Guy D. Eslick, Suzy M. Teutsch, Elizabeth J. Elliott

**Affiliations:** 1 Faculty of Medicine and Health, Speciality of Paediatrics and Child Health, The University of Sydney, Sydney, NSW Australia; 2 Australian Paediatric Surveillance Unit, Kids Research Sydney Children’s Hospitals Network, Westmead, NSW Australia

**Keywords:** Children, Parents, Psychological harms, Rare diseases

## Abstract

Rare childhood diseases can impose significant burden on the lives of affected children, their parents or primary caregivers, and their families. Evaluating the extent and scope of this burden is vital to yield reliable data to inform better support for families. The aim of this systematic review was to identify specific surveys and questionnaires used to assess the psychological harms of rare diseases on parents/primary caregivers and families, and to summarize the harms described by studies that have administered these surveys. MEDLINE, Embase, PsycINFO, and Google Scholar electronic databases were comprehensively searched in 2024 for published studies evaluating psychological harms on parents/primary caregivers and families caring for a child or young person aged 0–21 years and living with a rare disease, using both validated and non-validated surveys. Full text articles were screened and assessed by two independent reviewers. From the initial search, 350 studies were included after removing duplicates. Following full text review, 14 cross-sectional studies were included that used surveys to assess the psychological harms on parents/primary caregivers. Of the 22 surveys identified, 17 had been validated. Anxiety and stress were the most frequent and significant psychological conditions experienced by parents/primary caregivers of a child with a rare disease. More awareness of the psychological harms on parents and primary caregivers of children with rare diseases is needed to ensure they receive adequate support.

**Supplementary Information:**

The online version contains supplementary material available at 10.1007/s10567-025-00533-7.

## Introduction

Rare diseases often have their onset in childhood and are usually chronic and progressive conditions associated with delayed diagnosis (Dawkins et al., 2017). Most have a lifelong impact on the quality of life of the patient, their primary caregivers and family (Jaffe et al., 2010; Zurynski et al., 2008). Although individually rare, these diseases are together relatively common, affecting approximately 3.5–5.9% or 263–446 people globally (Nguengang Wakap et al., 2020). Although each rare disease is unique, the burdens they impose can be studied collectively due to the similar ways in which they affect children their families, parents, and primary caregivers (Anderson et al., 2013; Pelentsov et al., 2015).

High emotional strain and parental stress have been reported by those caring for a child with a rare disease. For instance, Anderson et al., (2013) found that more than three quarters of parents/primary caregivers of children with rare metabolic disorders experienced high levels of psychological stress. Moreover, in a survey of 564 caregivers of children with a range of rare diseases, over half reported that they had not been asked about their mental health (Spencer-Tansley et al., 2022). Thus, the impact of rare diseases on the mental health of parents/primary caregivers is insufficiently recognized (Dellve et al., 2006; Fuerboeter et al., 2021; Zurynski et al., 2008). While several literature reviews have assessed the quality of life and stress associated with caring for a child with a rare disease (Atkins & Padgett, 2024,; Boettcher et al., 2021a; Dias et al., 2023; Fitzgerald & Gallagher, 2022; Pavisich et al., 2024; von der Lippe et al., 2022; Wu et al., 2024), few have focused on specific psychological consequences, and none have identified the range of surveys used to assess the psychological harm on families and primary caregivers.

The primary objective of this study was to systematically review all available literature to identify the range and scope of surveys used in assessing the psychological harm on parents/primary caregivers of a child living with a rare disease. Other objectives included determining the number of validated and non-validated surveys used in the literature, identifying the main psychological harms assessed using validated surveys, and understanding the severity and patterns of adverse psychological effects from caring for a child with a rare disease.

## Method

### Study Design and Literature Search

This systematic review followed the Preferred Reporting Items for Systematic Reviews and Meta-Analysis (PRISMA) guidelines (Page et al., 2021). A comprehensive literature search was conducted on 30 May 2024 of MEDLINE, Embase, and APA PsycInfo electronic databases, using a detailed search strategy (see Supplementary material, Table S1). Assistance from a research librarian at The Children’s Hospital at Westmead in Sydney, Australia helped identify four key concepts for the search strategy: (1) rare diseases in children, (2) surveys, questionnaires, instruments and tools, (3) impacts or burdens, and (4) family and primary caregivers. As the focus of this review was harms on the psychological health of parents and caregivers, we did not include specific search terms that would identify psychological benefits to families, e.g., resilience. To find additional articles, a search was also performed in Google Scholar on 5 June 2024. If the validity and reliability status of a survey was unclear in an included article, relevant cited articles were also reviewed.

### Article Selection

Following the initial database searches, all citations found were imported into both EndNote citation management (Clarivate, Philadelphia, PA, USA) and Covidence systematic review (Veritas Health Innovation, Melbourne, Australia) software. Titles and abstracts were screened in Covidence, and duplicates were removed both automatically and manually. Full text articles for the remaining citations were imported into Covidence for review by two reviewers (LJB and GDE). Disagreements in the full text review were resolved by a third reviewer (SMT).

Eligible articles included those published in any language, and described quantitative or mixed study designs, study populations of parents or primary caregivers or families of children aged 0–21 years old who were diagnosed with a rare disease, any rare disease(s), all genders, validated and non-validated surveys, and impacts or burdens on children, adolescents, or young adults living with a rare disease, their siblings, parents or primary caregivers. Articles were excluded if they described adults with a rare disease (including those where the results for adults and children could not be separated), children with common diseases, qualitative studies, were editorials, conference abstracts, or review articles, did not have full text availability, or focused only on societal/healthcare outcomes.

### Quality Assessment

A modified Newcastle–Ottawa Scale (NOS) for Cross-sectional Studies (see Supplementary material, Fig. S1) was used to assess the methodological quality of each eligible article (Modesti et al., 2016). The comparability component, which assessed whether confounders were investigated, was excluded as it was generally not relevant to the type of articles; thus, only the ‘selection’ and ‘outcome’ of each article were evaluated, with maximum scores of five and three, respectively, giving a total score of eight. Articles received a score of one if they included a participant sample size of ≥ 30 and did not receive a score if the sample size was less than this.

### Data Extraction

Data were extracted from eligible articles into an MS-Excel spreadsheet, and included: author(s), publication year, geographic location (by continent), sample size, mean age, standard deviation, and self-reported gender distribution of parents/caregivers and children, rare disease(s) of children, name of survey, and psychological outcomes. Information was also extracted about the surveys used, including: the name of survey, subscales, number of items, Cronbach alpha coefficient, and test–retest reliability coefficient. A survey was classified as validated if this was explicitly stated in the study, if the study provided data demonstrating its validation, or the study cited a reference which provided this data. Markers for reliability in this systematic review included the internal consistency of survey items (measuring the same construct) and the consistency/stability of results over time (with the Cronbach alpha coefficient and test–retest correlation coefficient, respectively); these values were extracted from either the included study or cited validation study (Bolarinwa, 2015). If the study included an intervention, only the baseline data were used.

### Data Analysis—Descriptive Statistics

For included studies, all parent/primary caregiver and child demographics and clinical characteristics were reported as means and standard deviations for continuous numerical data, and as percentages for categorical attributes. The average reported mean ages and gender distributions across studies were pooled using the available data.

## Results

### Study Selection and Quality Assessment

**Fig. 1:**
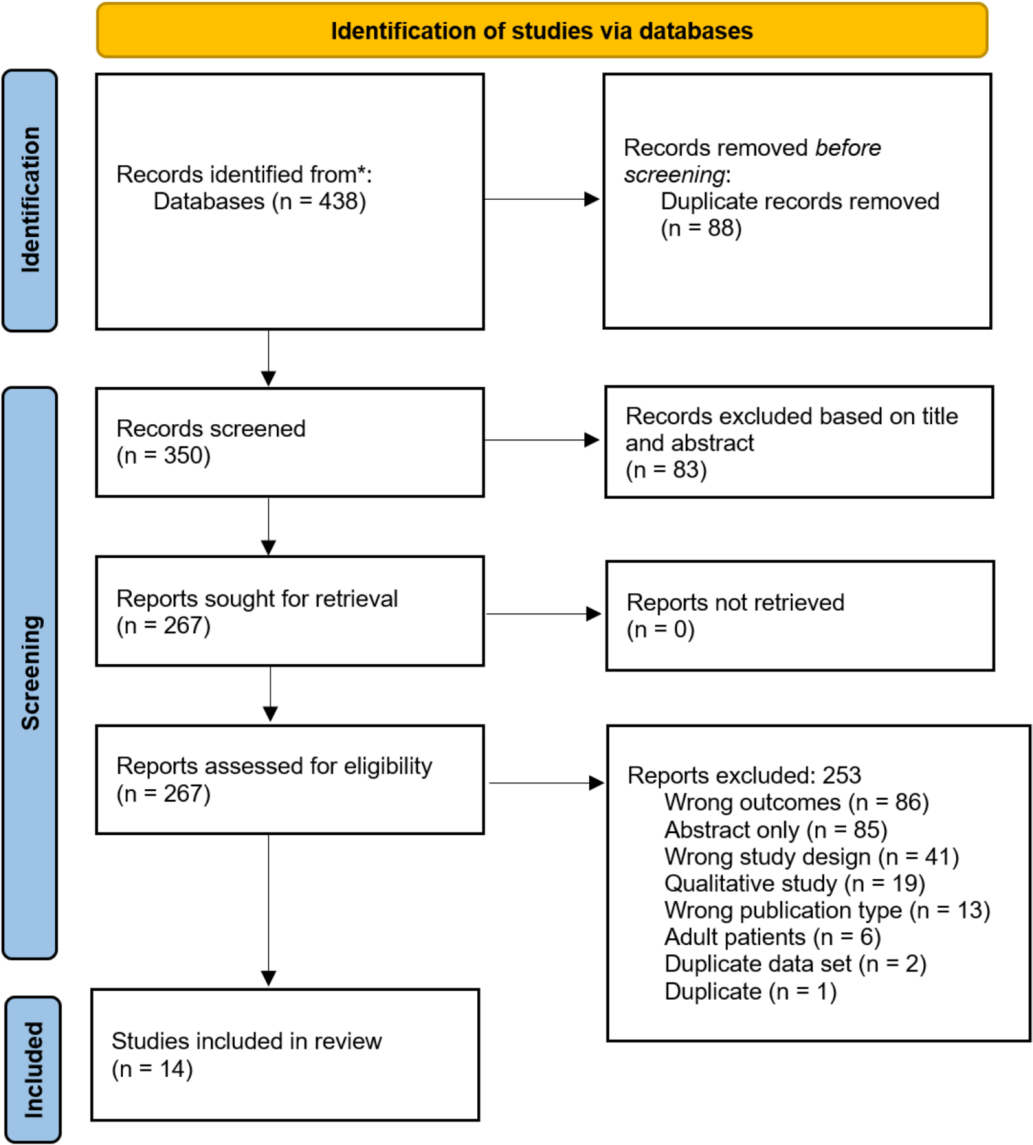
PRISMA flow diagram

From the literature search, 480 potential articles were identified, 350 were screened after removing duplicates and 267 underwent full text review. Fourteen articles were eligible for inclusion. The PRISMA flow diagram, constructed using Covidence software, is displayed in Fig. 1. Given the heterogenous nature of rare diseases studies, a meta-analysis was not conducted. Using a modified NOS, quality assessment of the 14 included articles showed that the mean score was 5.4 out of 8 (range: 4–7). The results of the quality assessment are presented in Table 1.

**Table 1:** Quality assessment scores of the 14 eligible articles using a modified Newcastle–Ottawa Scale for cross-sectional studies (refer to Supplementary material Table S1)

Studies	Year	Selection	Outcome	Total score
Adama et al.	2023	*****	**	7
Benedetto et al.	2023	**	**	4
Boettcher et al.	2020	****	**	6
Boettcher et al.	2021b	****	**	6
Bogart et al.	2016	***	**	5
Brown et al.	2017	**	**	4
Chu et al.	2022	****	**	6
Dellve et al.	2006	****	**	6
LoPresti et al.	2024	****	**	6
Michalak et al.	2022	***	**	5
Miodrag et al.	2015	***	**	5
Senger et al.	2016a	****	**	6
Tutus et al.	2024	****	**	6
Zajdel et al.	2022	***	*	4

### Article Characteristics

**Fig. 2:**
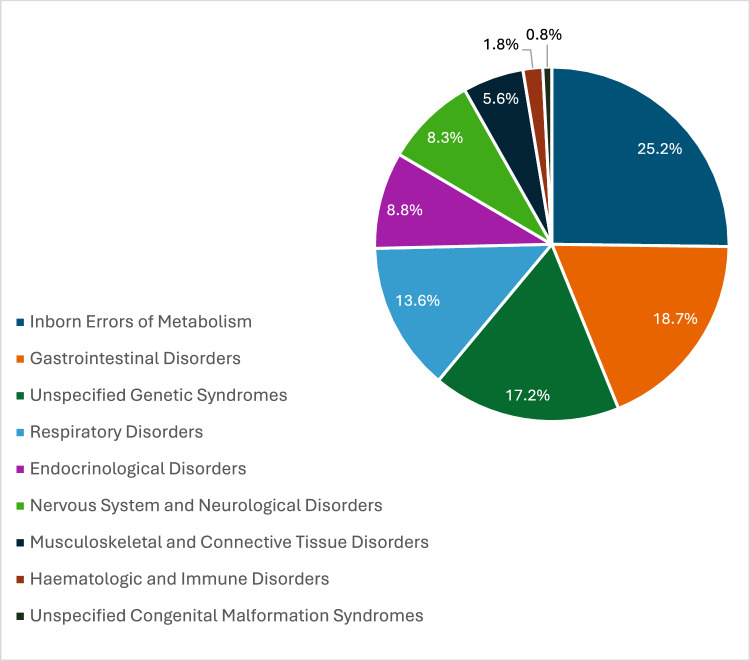
Distribution of rare disease types in 732 affected children in 12 of 14 articles* included in the review. *Two articles did not specify the number of affected children and were therefore not included in the pie chart

The characteristics of each included article are presented in Table 2. The articles originated from a diverse range of countries: six from Europe, four from North America, two from Asia, and two from Oceania. They were published between 2006 and 2024, the majority (64%) after 2020. All articles described cross-sectional studies assessing negative psychological impacts, i.e., harms and burdens on parents and caregivers of children living with rare diseases. Four articles evaluated the burdens of a specific rare disease on parents or primary caregivers and ten evaluated a mix of multiple rare diseases of different types but with similar clinical characteristics. The distribution of rare disease types in affected children is shown in Fig. 2. Twelve articles used surveys for parents or primary caregivers only, and two studies assessed both parents/primary caregivers and children.

**Table 2:** Characteristics of 14 articles included in the final systematic review, which assessed psychological impacts on parents and primary caregivers of a child with a rare disease

Author	Year	Continent, country	Sample size: children with rare disease Parents/primary caregivers	Female % of children with rare disease	Female % of parents/primary caregivers	Mean age in years (SD)	Rare disease(s)	Survey Name (validation study cited: yes/unknown)	Validation study	Psychological outcomes
Adama et al.	2023	Oceania, Australia	41 41 mothers	46.3	100	Parents: NR; Children: 10.61 (3.22)	Multiple	Parent Stigma Scale (Yes) Strengths and Difficulties Questionnaire (Yes)	Parents of children with chronic epilepsy and new-onset seizures, United States (Austin et al., 2004) Representative national sample of parents of children aged 5–15 years, United Kingdom (Goodman, 2001)	50% of parents/caregivers recorded perceived stigma about their child’s rare disease
Benedetto et al.	2023	Europe, Italy	14 12 mothers, 2 fathers	64.29	85.71	Parents: NR; Children: 7.93 (4.10)	Pompe Disease	Psychological Needs and Disease-Related Issues (Italian version) (Yes)	Samples of parents of children with autism or Down Syndrome (Benedetto et al., 2012); Samples of parents of children aged 0–18 years with leukemia or Hodgkin’s disease (Benedetto et al., 2022)	21% of mothers said they suffered"serious burnout"
Boettcher et al.	2020	Europe, Germany	95 72 mothers, 38 fathers	34.7	65.45	Mothers: 40.1 (7.38); Fathers: 43.1 (7.38); Children: 9.5 (5.48)	Central respiratory disorders; Restrictive ventilatory disorders; Obstructive ventilatory disorders	Brief Symptom Inventory (German version)	Sample of German patients aged 12–77 years attending clinics for treatment of anxiety disorders (Geisheim et al., 2002)	Mothers: severe depression and anxiety; fathers: mild depression and anxiety Fathers were working, mothers took on the caring role
Boettcher et al.	2021b	Europe, Germany	104 109 mothers, 101 fathers	38.6	51.9	Mother: 37.4 (5.98); Fathers: 40.3 (6.15); Children: 4.2 (3.33)	Rare congenital disorders requiring surgery	Brief Symptom Inventory (German version) (Yes) Strength and Difficulties Questionnaire (German version) (Yes)	Sample of German patients aged 12–77 years attending clinics for treatment of anxiety disorders (Geisheim et al., 2002) Representative population ample of parents of children aged 6–16 years, Germany (Klasen et al., 2003)	Psychological distress was severe in mothers and mild in fathers Mothers more involved in caring for their child
Bogart et al.	2016	North America, United States	NR 48	51.7	89.7	Parents: 43.69 (10.74); Children: 11.81 (10.40)	Moebius Syndrome	Hospital Anxiety and Depression Scale (Yes) Perceived Stigma Questionnaire (Yes)	Sample of general medical clinic outpatients aged 16–65 years with a range of health issues, United Kingdom (Zigmond & Snaith, 1983) Sample of parents of pediatric burn survivors (Lawrence et al., 2011)	Parents/caregivers recorded severe anxiety and depression Parents/caregivers recorded mild perceived stigma
Brown et al.	2017	Oceania, Australia	22 22	27.27	NR	NR	Inherited Metabolic Disorders	Kessler 10 Psychological Distress Scale (Yes) Parent Experience of Childhood Illness (Yes) Strengths and Difficulties Questionnaire (Yes)	Large representative population samples, United States (Kessler et al., 2002) Sample of parents of pediatric patients (age < 1–17 years) diagnosed with a brain tumor, United States (Bonner et al., 2006) Representative national sample of parents of children aged 5-15 years, United Kingdom (Goodman, 2001)	Parents/caregivers recorded severe guilt and worry, mild sorrow and anger Parents/caregivers recorded low levels of depression
Chu et al.	2022	Asia, Taiwan	77 100	36.36	58	Female caregivers: 42.4 (12.2); Male caregivers: 44.9 (11.0); Children: 9.9 (5.9)	Multiple	Center for Epidemiological Studies Depression Scale (Short Form) (Chinese version) (Yes)	National representative sample of adults aged ≥ 60 years enrolled in longitudinal study of aging, Taiwan (Chiao et al., 2011)	Mothers twice as likely to experience depression than fathers
								Pediatric Inventory for Parents (Chinese version) (No)	–	Depression in mothers was severe and in fathers was mild
Dellve et al.	2006	Europe, Sweden	142 136 mothers, 108 fathers	60.56	55.74	Parents: NR; Children: Median = 7	Multiple	Parenting Stress Index (Swedish version) (Yes)	Population samples of mothers of young children aged ≥ 6 months to 3 years, Sweden (Östberg et al., 1997)	Mothers recorded severe stress; fathers recorded mild stress
LoPresti et al.	2024	Asia, Japan	36 36	47	91.67	Children: 12.4 (8.8)	Rare drug-resistant epilepsy including Lennox-Gastaut syndrome; Dravet syndrome; tuberous sclerosis complex-associated epilepsy	Hospital Anxiety and Depression Scale (Yes)	Sample of general medical clinic outpatients aged 16–65 years with a range of health issues, United Kingdom (Zigmond & Snaith, 1983)	Parents/caregivers recorded severe anxiety and mild depression
Michalak et al.	2022	Europe, Poland	35 35	31.4	NR	Children: 12.3 (3.6)	Pediatric Monogenic Diabetes due to GCK Mutation	The World Health Organization Five Well-Being Index (assumed to be in Polish language) (Unknown)	–	Parents/caregivers recorded increased levels of anxiety, depression, but decreased stress compared to those caring for children with more common type 1 diabetes
								Beck Depression Inventory (assumed to be in Polish language) (Unknown)	–	
								Parental Stress Index (assumed to be in Polish language) (Unknown)	–	
								Stress Index for Parents of Adolescents (assumed to be in Polish language) (Unknown)	–	
Miodrag et al.	2015	North America, United States	IC defects: 11, UPD: 14 NR	NR	NR	IC defects (Mothers): 38.60 (10.02); IC defects (Fathers): 41.40 (8.99); UPD (Mothers): 39.29 (6.42); UPD (Fathers): 42.32 (6.45); IC defects (Children): 5.39 (5.34); UPD (Children): 5.83 (4.14)	Angelman syndrome	Parenting Stress Index (long form) (Yes)	Large population of parents of children aged 6 months–12 years, United States (Abidin, 1995)	Parents/caregivers recorded mild stress
Senger et. al	2016a	North America, United States	NR 231	51	95	Parents: 42 (8.30)	Mitochondrial Disease	Parent Experience of Child Illness (Yes) Pediatric Inventory for Parents (Yes)	Sample of parents of pediatric patients (age < 1–17 years) diagnosed with a brain tumor, United States (Bonner et al., 2006) Parents of children and adolescents followed up by a hospital oncology clinic, United States (Streisand et al., 2001)	Parents/caregivers recorded moderate guilt and worry, mild sorrow and anger, and increased stresses related to caring for children
Tutus et. al	2024	Europe, Germany	36 30 mothers, 6 fathers	38.89	83.33	Parents: 40.47 (8.02); Child: 7.21 (5.61)	Multiple	Generalized Anxiety Disorder Questionnaire-7 (German version) (Yes) Fear of Progression Questionnaire short form (German version (Yes) Patient Health Questionnaire-9 (German version) (Yes)	Large national representative population sample aged ≥ 14 years, Germany (Löwe et al., 2008) Large sample of breast cancer patients, Germany (Mehnert et al., 2006) Samples of medical and psychosomatic outpatients, Germany (Gräfe et al., 2004)	Parents/caregivers recorded moderate anxiety and depression, and severe fear about the future
Zajdel et. al	2022	North America, United States	105 223	43.8	69.1	Caregivers: 44.03 (12.48); Children: 11.2 (10.4)	Multiple	Perceived Stress Scale (Yes)	Two samples of college students and one of adults enrolled in a smoking cessation program, United States (Cohen et al., 1983)	Parents/caregivers recorded moderate stress

*NR* not reported, *N/A* not applicable, *IC* imprinting center, *SD* standard deviation, *UPD* paternal uniparental disomy

The study sample size of children with a rare disease ranged from 14 to 142, and for parents/primary caregivers sample sizes ranged from 14 to 244. The total pooled sample size was 732 for children with a rare disease (from 12 studies) and 1350 for parents/primary caregivers (from 14 studies). The pooled mean age for children was 9 years (from 9 studies), and 42 years for parents/primary caregivers (from 7 studies). The pooled gender distribution among children (from 10 studies) showed that the majority (53.4%) were male, and the majority (70.2%) of parents and primary caregivers were female (based on data from 11 studies).

### Psychological Surveys Used in Rare Disease Studies

Table 2 shows the psychological surveys used in each of the included articles. Table 3 shows the characteristics of the surveys used in these studies, as described in the original context in which they were validated. Of the 22 surveys identified, 17 (77%) had undergone reliability and validity testing. Of the 21 surveys where the number of items was reported, seven contained 10 items or less and six contained more than 30 items. Among the 15 validated surveys used to assess the burden on parents and primary caregivers, only nine (60%) were tested for reliability using parent samples, including the Parent Experience of Childhood Illness (PECI) (Bonner et al., 2006), Parent Stigma Scale (PSC) (Austin et al., 2004), Parenting Stress Index (long form) (Abidin, 1995), Parenting Stress Index (Swedish version) (Östberg et al., 1997), Pediatric Inventory for Parents (PIP) (Streisand et al., 2001), Perceived Stigma Questionnaire (Lawrence et al., 2011), Strengths and Difficulties Questionnaire (Goodman, 2001), Strengths and Difficulties Questionnaire (German version) (Klasen et al., 2003), and Psychological Needs and Disease-Related Issues (Italian version) (Benedetto et al., 2012, 2022). The content of only ten of the 17 validated surveys was able to be accessed, and examination showed that the majority of them included generic questions, i.e., they were not specific to any disease, rare or otherwise.

**Table 3:** Surveys (*n* = 22) used in 14 eligible articles assessing psychological impacts on parents and primary caregivers of a child with a rare disease

Name of survey	No. of studies that used survey	Subscales	Number of items	Interpretative guidelines and clinical cut-off values/	Cronbach alpha coefficient	Test–retest correlation coefficient	Validation verified (yes/no)
Beck Depression Inventory (assumed to be in Polish language)	1	NR	21	Higher scores indicate increased severity of depression	NR	NR	No
Brief Symptom Inventory (German version)	2	Somatization; Obsession–compulsion; Interpersonal sensitivity; Depression; Anxiety; Hostility; Phobic anxiety; Paranoid ideation; Psychoticism; Overall distress	53	Higher scores indicate increased psychological distress	0.70–0.87	NR	Yes
Center for Epidemiological Studies Depression Scale Short Form (Chinese version)	1	NR	10	Scores 0–30 represent increasing levels of depressive symptoms, with scores ≥ 10 indicating depression	0.78–0.87	NR	Yes
Fear of Progression Questionnaire Short Form (German version)	1	NR	12	Total scores between 12 and 60 indicate increasing fear of progression Cut-off score of 34 represents dysfunctional fear of progression	0.87	NR	Yes
General Anxiety Disorder Scale-7 (German version)	1	N/A	7	Scores 0–21, score of 5 represents mild, 10 moderate, and 15 severe anxiety	0.89	NR	Yes
Hospital Anxiety and Depression Scale (self-report)	2	Depression; Anxiety	14	Scores 0–21 represent increasing levels of depression and anxiety	0.50–0.80	0.68–0.72	Yes
Kessler 10 Psychological Distress Scale	1	N/A	10	Low (10–15), moderate (16–21, high (2–30), very high (31–50) levels of distress	0.93	NR	Yes
Parent Experience of Childhood Illness	2	Emotional Resources; Guilt and Worry; Unresolved Sorrow and Anger; Long-term Uncertainty	25	Higher scores in 1 st domain indicate positive feelings; higher scores in other domains indicate negative feelings	0.72–0.89	NR	Yes
Parent Stigma Scale	1	N/A	5	Higher scores indicate increased perceived stigma	0.77–0.79	NR	Yes
Parenting Stress Index (assumed to be in Polish language)	1	Depression Attachment Role restriction Competence Isolation Spouse/Parenting Partner Relationship Health	NR	Higher scores indicate increasing levels of perceived stress	NR	NR	No
Parenting Stress Index Long Form	1	Child domain: Distractibility/Hyperactivity; Adaptability; Reinforces parent; Demandingness; Mood; Acceptability; Parent domain: Competence; Isolation; Attachment; Health; Role restriction; Depression; Spouse	101	Higher scores indicate increasing levels of perceived stress	0.95	0.65–0.96	Yes
Parenting Stress Index (Swedish version)	1	Incompetence; Social isolation; Role restriction; Spouse relationship problems; Health problems	34	Higher mean scores indicate increasing levels of perceived stress	0.57–0.90	0.79–0.89	Yes
Patient Health Questionnaire-9 (German version)	1	NR	9	Scores 0–27. Higher scores indicate increasing levels of depression. Scores of 5 represent mild, 10 moderate, 15 moderately severe, and 20 severe depression	0.79–0.88	0.60	Yes
Pediatric Inventory for Parents	2	Communication; Medical care; Emotional functioning; Role constraints	42	Higher scores indicated increased levels of perceived stress	0.95–0.96	NR	Yes
Pediatric Inventory for Parents (Chinese version)	2	Communication; Medical care; Emotional functioning; Role constraints	42	Higher scores indicated increased levels of perceived stress	NR	NR	No
Perceived Stigma Questionnaire (parent-reported)	1	Hostile behavior; Confused/staring behavior; Absence of friendly behavior	21	Higher scores indicate increased perceived stigma	NR	NR	Yes
Perceived Stress Scale	1	N/A	10	Higher scores indicate increased levels of perceived stress	0.84–0.86	0.85	Yes
Psychological Needs and Disease-Related Issues (Italian version)	1	Uncertainty about disease; Resources; Fears for the child; Fears for themselves; Fears for siblings	37	Higher scores indicate greater perceived needs and disease-related issues	0.68–0.94	NR	Yes
Strengths and Difficulties Questionnaire—parent-reported extended version for children and adolescents	2	Hyperactive measure; Emotional problems; Peer problem; Conduct problems; Prosocial behaviors	25	Scores of 0–10 for each of five domains. Increased scores for the four difficulties domains scores indicate worse mental health, while increased score for prosocial behaviors indicates increased strength	0.73	0.62	Yes
Strengths and Difficulties Questionnaire (German version)	1	Hyperactive measure; Emotional problems; Peer problem; Conduct problems; Prosocial behaviors	25	Scores of 0–10 for each of five domains. Increased scores for the four difficulties domains scores indicate worse mental health, while increased score for prosocial behaviors indicates increased strength	0.82	NR	Yes
Stress Index for Parents of Adolescents (assumed to be in Polish language)	1	Adolescent-related domain; Moodiness; Withdrawal; Delinquency; Failure to achieve; Parent-related domain; Life restrictions; Relationship with spouse/partner; Social alienation; Guilt	112	Increasing scores indicate higher levels of perceived stress	NR	NR	No
The World Health Organization Five Well-Being Index (assumed to be in Polish language)	1	N/A	5	Scores 0–25. Higher scores indicate increased perceived wellbeing	NR	NR	No

*NR* not reported, *N/A* not applicable

Six validated surveys assessed the level of parent or primary caregiver anxiety and/or depression in study participants: the Hospital Anxiety and Depression Scale (HADS) (Zigmond & Snaith, 1983), the Kessler 10 Psychological Distress Scale (Kessler et al., 2002), the Centre for Epidemiological Studies Depression Scale (CES-D) Short Form (Chinese version) (Chiao et al., 2011), the PHQ-9)(German version) (Gräfe et al., 2004), and GAD-7 (German version) (Löwe et al., 2008). Additionally, the Brief Symptom Inventory (BSI) (German version) included nine subscales to investigate multiple psychological conditions, with two assessing anxiety and depression (Geisheim et al., 2002). The German version of GAD-7 was developed with seven steps of translation and blind-back translation and reflected DSM-IV criteria (Löwe et al., 2008). Similarly, the 9 items of the German version of PHQ-9 reflected each of the 9 DSM-IV criteria (Gräfe et al., 2004).

Four validated surveys were used to measure parent or primary caregiver stress: the PIP (Streisand et al., 2001), PSI (Swedish version) (Östberg et al., 1997), PSI Long Form (Abidin, 1995), and the Perceived Stress Scale (PSS) (Cohen et al., 1983). The PSI Long Form assessed the perceived stress considering both child and parent factors (Abidin, 1995). Validated surveys used to investigate health-related stigma include the Parent Stigma Scale (PSS) (Austin et al., 2004) and the Perceived Stigma Questionnaire (PSQ) (Lawrence et al., 2011). Also, the validated Parent Experience of Childhood Illness (PECI) Short Form was used to assess overall emotional functioning in study participants in two studies (Bonner et al., 2006). Furthermore, in one study, the FoP-Q-SF was used to measure parental fear of disease progression (Mehnert et al., 2006). Interestingly, the Psychological N-DRI was the only survey that included a domain to assess the psychological harms on siblings of a child with a rare disease (Benedetto et al., 2012).

Surveys for which validation status was unclear, or when it was not possible to determine whether they had been tested for reliability and validity were identified and included: the Beck Depression Inventory (BDI) (assumed to be in Polish language) (Michalak et al., 2022), the Parenting Stress Index (PSI) (assumed to be in Polish language) (Michalak et al., 2022), the Pediatric Inventory for Parents (Chinese version) (Chu et al., 2022), the Stress Index for Parents of Adolescents (SIPA) (assumed to be in Polish language) (Michalak et al., 2022), and the World Health Organization Five Well-Being Index (assumed to be in Polish language) (Michalak et al., 2022).

### Psychological Outcomes Assessed in Parents/Primary Caregivers

Among the 14 studies, seven studies assessed in parents and primary caregivers the presence of depression and/or anxiety, five measured the level of stress, two assessed aspects of emotional functioning, and two investigated perceptions of stigma associated with their child’s rare disease(s).

The included studies used a variety of surveys to assess the psychological harm of either parents-only, children and parents, or primary caregivers-only. In three studies, primary caregivers were the sole participants and comprised mainly of parents: 97% (LoPresti et al., 2024), 90% (Chu et al., 2022), and 69.4% (Zajdel et al., 2022), respectively. The second most common primary caregivers were grandparents, followed by other family members including siblings, aunts, and uncles (Zajdel et al., 2022).

Four studies assessed the psychological harm of a child’s rare disease on mothers and fathers separately (Boettcher et al., 2020, 2021b; Chu et al., 2022; Dellve et al., 2006). These studies found that mothers experienced greater stress, anxiety, and/or depression compared to fathers. Additionally, four studies reported that primary caregivers were mostly mothers, and Dellve et al. (2006) found only 24% of mothers had full-time jobs, compared to 87% of fathers (Boettcher et al., 2020, 2021b; LoPresti et al., 2024; Zajdel et al., 2022).

Specific psychological outcomes, including depression, anxiety, stress, stigma, and emotional functioning in parents/primary caregivers and children, as measured using validated surveys (Table 2), are described in the following sections. The interpretative guidelines/clinical cut-off values for each survey are listed in Table 3.

#### Depression and Anxiety

Seven studies measured anxiety and/or depression: six found normal to low levels of anxiety and/or depression in most parents/primary caregivers (Boettcher et al., 2020; Bogart & Hemmesch, 2016; Brown et al., 2017; Chu et al., 2022; LoPresti et al., 2024; Tutus et al., 2024), one did not explicitly report these outcomes (Boettcher et al., 2021b). However, Tutus et al. (2024) reported moderate levels of both anxiety and depression using the GAD-7 and PHQ-9 surveys. In addition, Boettcher et al. (2020) found that 9.7% and 16.7% of females exhibited clinically significant anxiety or depression, respectively. Similarly, using the Kessler 10 Psychological Distress Scale, Brown et. al. (2017) identified moderate to very high levels of distress in 27% of parents of children with inherited metabolic disorders.

Using the BSI survey, Boettcher et al. (2020) found that on average mothers had anxiety scores 2.2 times higher and depression scores 1.7 times higher than fathers. The study noted that over 70% of mothers were primary caregivers. Similarly, Chu et al. (2022) reported a mean score of depressive symptoms which was 1.8 times higher in females than males using the CES-D survey (Chinese version).

Two other studies reported a higher severity of anxiety symptoms than depression (Bogart & Hemmesch, 2016; LoPresti et al., 2024). LoPresti et al. (2024), using the HADS survey to assess primary caregivers of children with severe drug-resistant epilepsy, reported a mean anxiety score approximately twice as high in those providing more than 21 h of care per week than those providing less than this amount.

#### Stress

Five studies assessed stress among parents/primary caregivers. Firstly, Chu et al. (2022) found that female primary caregivers reported more parenting stress and provided more hours of care each day. Secondly, with the PSI (Swedish version), Dellve et al. (2006) found that mothers had more stress than fathers, with both having more stress compared to a comparison population group of randomly selected parents of children less than 3.5 years old. In addition, Boettcher et al. (2020) reported that 25% of mothers and 7.9% of fathers had a clinically significant score of overall distress, when the Global Severity Index was calculated from the BSI responses.

Using the PSI survey (long form), Miodrag and Peters (2015) assessed parents of children with different molecular subtypes of Angelman syndrome and found 100% and 64.3% of parents who had children with IC defects and UPD subtypes, respectively, also had clinical levels of child-related stress. Dellve et al. (2006) also reported higher levels of stress in parents of children with behavior-related rare diseases.

Senger et al. (2016a) found higher PIP survey scores, indicating higher levels of stress, in parents caring for a child with mitochondrial disease than Chu et al. (2022), who surveyed a mixed sample of rare diseases and more common genetic diseases. In both studies, the ‘frequency’ subscale score was higher than the ‘difficulty’ subscale scores.

#### Emotional Functioning

In Senger et al. (2016a), parents of children with mitochondrial disease reported mean scores for guilt and worry, and sorrow and anger on the PECI survey within the ‘sometimes’ to ‘often’ range. Likewise, Brown et al. (2017) found slightly elevated mean scores in parents of children with inherited metabolic disorders, within the ‘rarely’ to ‘sometimes’ range; although, most parents in this study did not show elevated psychological distress relative to Australian normative data.

Two studies investigated parents’ perception of health-related stigma and found that parents reported a moderate to strong perception of stigma toward their child (Adama et al., 2023; Bogart & Hemmesch, 2016).

## Discussion

This is the first systematic review to assess the use and scope of surveys that measure the psychological harms on parents, primary caregivers and families caring for a child living with a rare disease. This review verified that 77% of surveys used in included studies were validated, and the main psychological conditions and consequences assessed were: anxiety, depression, stress, stigma, and emotional functioning.

Anxiety and stress were the most frequent self-reported psychological harms by parents and primary caregivers of children with a rare disease (Boettcher et al., 2020; Bogart & Hemmesch, 2016; Brown et al., 2017; Chu et al., 2022; Dellve et al., 2006; LoPresti et al., 2024; Miodrag & Peters, 2015; Senger et al., 2016a; Tutus et al., 2024). Mothers tended to report more significant psychological difficulties and worse mental health outcomes than fathers, including higher levels of anxiety, depression, and stress (Boettcher et al., 2020, 2021b; Chu et al., 2022; Dellve et al., 2006). These findings are consistent with results of two other reviews which found that mothers typically experienced worse mental health outcomes than fathers (Atkins & Padgett, 2024; Malm-Buatsi, et al., 2015). This may reflect the fact that mothers most often assume the role of primary caregiver in families and fathers are the primary provider, potentially increasing the psychological burden for mothers (von der Lippe et al., 2022). Our findings differ however from two previous reviews evaluating parental quality of life and stress, which found no significant gender differences in psychological outcomes in the majority of studies (Boettcher et al., 2021a; Fitzgerald & Gallagher, 2022). However, given that most respondents were mothers in the included studies, more research is needed to adequately assess psychological harms on fathers.

Some evidence suggests that parents/primary caregivers of children with a rare disease, such as mitochondrial diseases, experience higher levels of stress than those of children with more common chronic illnesses (Senger et al., 2016b). Furthermore, rare diseases with certain characteristics cause more stress among parents/primary caregivers than others; two studies highlighted the positive association between rare conditions associated with challenging behaviors, such as Angelman syndrome and Cornelia de Lange syndrome, and the level of parental stress (Dellve et al., 2006; Miodrag & Peters, 2015). Additionally, of the two studies using the PIP survey for evaluating stress, one found higher stress in a cohort of parents of children with mitochondrial disease (Senger et al., 2016a) than in the other, which described mostly rare diseases (some of the genetic diseases were not rare but could not be differentiated in the results) (Chu et al., 2022). This supports findings from Fitzgerald and Gallagher. (2022), that certain syndrome-specific phenotypes are associated with more parental adversity than other rare diseases. Reasons for high parental stress may be the uncertain prognosis of many rare diseases, social isolation, and financial concerns (Pelentsov et al., 2015; Senger et al., 2016a). This underscores the importance of measuring the impacts of children’s rare diseases on their parents/primary caregivers to determine the level of unmet needs among parents/primary caregivers and the way in which we should respond. A lack of parental support may be the result of inadequate knowledge and awareness among healthcare professionals about how parents are affected, a gap in communication between healthcare professionals and parents, a paucity of support groups for certain rare diseases and the geographical locations of these groups (Pelentsov et al., 2016; von der Lippe et al., 2022).

Most (10 of 14) of the studies in this review investigated the psychological burden on parents and primary caregivers in the context of multiple rare diseases, rather than an individual rare disease. However, given that sample size is a common limitation in single rare disease studies, the outcomes of future research may be more relevant if participants are recruited from similar clinical categories (e.g., respiratory diseases or inherited metabolic diseases).

This review shows that research on the burden of rare diseases on the mental health of parents and primary caregivers has increased in recent years, with a significant number of studies published after 2020 (Adama et al., 2023; Benedetto et al., 2023; Boettcher et al., 2021b; Chu et al., 2022; LoPresti et al., 2024; Michalak et al., 2022; Tutus et al., 2024; Zajdel et al., 2022). Moreover, Anderson et al. (2013) noted that few validated surveys had been used in previous research to assess the burden of rare diseases on families. In contrast, validated surveys assessing psychological harms were used in approximately 80% of studies included in our review. This approach of using validated surveys is essential for capturing meaningful data on the burden of rare diseases on families and to improve the support they receive (Anderson et al., 2013; Boettcher et al., 2021a).

It was uncertain from available information whether the remaining 23% of surveys in studies identified in this review were validated. Furthermore, only one study tested the reliability of the surveys used in the specific sample of parents of children with a rare disease described (Tutus et al., 2024). No other surveys used in the 14 studies were tested in rare disease populations, and only 60% of all surveys assessing parents/primary caregivers had been validated in a parent population. A closer examination of several surveys revealed that most were generic in nature and could therefore be used to study a range of rare diseases. Most surveys contained multiple questions; however, it is important for surveys to be as brief as possible while preserving their reliability and validity, so as not to burden families and increase compliance. To enhance the relevance of surveys for rare disease research, more should be validated in children with rare diseases and in their parents.

We acknowledge that all the studies we identified were conducted in high-income countries and there were no studies originating from countries on the African continent. Conducting studies in a wider range of geographic settings, including low to middle income countries, will allow a more complete assessment of psychological harms and burdens experienced by parents and caregivers of children living with rare diseases and increase generalizability of the results. Furthermore, it is important for surveys to be validated within the specific population(s) that there are administered to, in order to minimize anomalies due to cultural differences.

## Strengths and Limitations

This study has several strengths, including the systematic identification, from a comprehensive search of multiple databases, of validated surveys assessing psychological harms of a wide range of childhood rare diseases on parents and primary caregivers. However, there are also some limitations. Firstly, this review only focused on studies using surveys to assess negative psychological impacts, i.e., burdens and harms of caring for a child with a rare disease, such as stress, depression, and anxiety and did not include studies using surveys to assess the full range of impacts that may occur, including positive impacts such as resilience and coping (Manalel et al., 2024; Neumann et al., 2022, 2024; Picci et al., 2015). Secondly, studies using validated quality of life surveys with a psychological component were excluded to keep scope of the review focused, which means additional psychological harms may have been missed. Also, multiple validation studies of the same survey may have been conducted in different populations, however the validation characteristics described in this review were verified only from the data reported in the included studies and the cited validation studies. Many studies did not provide a clear description of both the validity and reliability of the surveys used. To improve the quality of research in this field, future studies should describe these characteristics for all surveys used and specify whether they were validated within similar populations. Moreover, only one study asked participating parents and primary caregivers about their own medical and psychological history, which is likely relevant when investigating symptoms of anxiety and depression and may have biased study results. Articles on children with undiagnosed diseases were omitted from this study, so additional psychological harms in those groups were not evaluated. Also, it is possible that the literature search missed relevant publications that addressed the psychological burdens in specific rare diseases that had not been identified as rare in the article’s title or key words. Finally, biases may have occurred in self-reported surveys of burdens on parents and primary caregivers and in parent-reported surveys of impacts on their children.

## Conclusion

The findings from this study show that anxiety and stress are significant psychological harms reported by parents and primary caregivers of a child with a rare disease. Future studies should include validation characteristics of surveys used and consider the prior psychological history of parents and primary caregivers. Utilizing a combination of surveys would potentially provide a more thorough assessment of parental psychological well-being. Including a sample of children with rare diseases with similar clinical characteristics (e.g., rare metabolic diseases) may also be beneficial to assist in understanding how different disease groups differentially impact psychological harms in parents and primary caregivers and hence determine their care needs.

To gain a more comprehensive and in-depth understanding of the impacts of children’s rare disease on all family members, future studies should use psychological-focused surveys to assess the mental wellbeing and harms of siblings. The scope of this systematic review of validated surveys could also be extended to other types of impacts of rare diseases on parents and primary caregivers, such as quality of life, financial, and social impacts. Finally, it is important that healthcare professionals managing children with rare diseases recognize that parents and primary caregivers need appropriate psychological supports, and to incorporate these into the holistic management of their patients.

## Supplementary Information

Below is the link to the electronic supplementary material.

Supplementary file1 (DOCX 21 KB)

## Data Availability

No datasets were generated or analyzed during the current study.
