# Supplementary material for: Assessing Psychological Harms on Parents and Primary Caregivers of Children Living with a Rare Disease: A Systematic Review of the Scope and Validity of Surveys Utilized
Source: Clin Child Fam Psychol Rev. 2025 Jun 30;28(3):612–30. doi: 10.1007/s10567-025-00533-7 (PMC12634755; doi:10.1007/s10567-025-00533-7)
Supplement: Supplementary file 1 — Supplementary file1 (DOCX 21 KB) [file 10567_2025_533_MOESM1_ESM.docx]

**Supplementary Material**

**Table S1** Search strategy used in each of the databases

| **Database** | **Search String** |
| --- | --- |
| MEDLINE | (Rare Diseases/ OR (rare disease* OR rare condition* OR rare disorder*).tw. OR orphan disease*.tw.) AND  limit (to "all child (0 to 18 years)") AND  (exp "Surveys and Questionnaires"/ OR Survey*.tw. OR Questionnaire*.tw. OR instrument*.tw. OR tool*.tw.) AND  (exp "Quality of Life"/ OR quality of life*.tw. OR ((economic OR financial OR monetary) adj3 (impact* OR burden* OR strain* OR stress* OR cost* OR hardship* OR challenge* OR fatigue OR pressure* OR demand*)).tw. OR ((Psychological OR emotional OR mental OR social) adj3 (impact* OR burden* OR strain* OR stress* OR cost* OR hardship* OR challenge* OR fatigue OR pressure* OR demand*)).tw. OR ((health-related OR physical OR education OR family) adj3 (impact* OR burden* OR strain* OR stress* OR cost* OR hardship* OR challenge* OR fatigue OR pressure* OR demand*)).tw.) AND  (exp Family/ OR exp Caregivers/ OR (family OR families OR mother* OR father* OR sibling* OR brother* OR sister* OR parent* OR caregiver* OR carer*).tw.) |
| Embase | (exp rare disease/ OR (rare disease* OR rare condition* OR rare disorder*).tw. OR orphan disease*.tw.)  AND  ((exp questionnaire/ OR survey*.tw. OR questionnaire*.tw. OR instrument*.tw. OR tool*.tw.)  AND  (exp "quality of life"/ OR quality of life*.tw. OR ((economic OR financial OR monetary) ADJ3 (impact* OR burden* OR strain* OR stress* OR cost* OR hardship* OR challenge* OR fatigue OR pressure* OR demand*)).tw. OR ((Psychological OR emotional OR mental OR social) ADJ3 (impact* OR burden* OR strain* OR stress* OR cost* OR hardship* OR challenge* OR fatigue OR pressure* OR demand*)).tw. OR ((health-related OR physical OR education OR family) ADJ3 (impact* OR burden* OR strain* OR stress* OR cost* OR hardship* OR challenge* OR fatigue OR pressure* OR demand*)).tw.))  AND  (exp family/ OR exp caregiver/ OR (family OR families OR mother* OR father* OR sibling* OR brother* OR sister* OR parent* OR caregiver* OR carer*).tw.)  AND  limit to child |
|  | ((rare disease* OR rare condition* OR rare disorder*).tw. OR orphan disease*.tw.) AND  ((survey*.tw. OR questionnaire*.tw. OR instrument*.tw. OR tool*.tw. OR exp questionnaire/) AND  (exp "quality of life"/ OR quality of life*.tw. OR ((economic OR financial OR monetary) adj3 (impact* OR burden* OR strain* OR stress* OR cost* OR hardship* OR challenge* OR fatigue OR pressure* OR demand*)).tw. OR ((Psychological OR emotional OR mental OR social) adj3 (impact* OR burden* OR strain* OR stress* OR cost* OR hardship* OR challenge* OR fatigue OR pressure* OR demand*)).tw. OR ((health-related OR physical OR education OR family) adj3 (impact* OR burden* OR strain* OR stress* OR cost* OR hardship* OR challenge* OR fatigue OR pressure* OR demand*)).tw.)) AND  (exp family/ OR exp caregivers/ OR (family OR families OR mother* OR father* OR sibling* OR brother* OR sister* OR parent* OR caregiver* OR carer*).tw.) AND  limit to (childhood OR adolescence) |
| Google Scholar | Rare disease + impacts + family + parent + survey + questionnaire |

**Figure S1** Modified Newcastle-Ottawa Scale for cross-sectional studies (maximum score = 8)

Selection: (Maximum 5 scores)

1. Representativeness of the cases:

a) Truly representative of the patients (consecutive or random sampling of cases). (1 score)

b) Somewhat representative of the average patients (non-random sampling). (1 score)

c) Selected demographic group of users. (0 score)

d) No description of the sampling strategy. (0 score)

1. Sample size:

a) Justified and satisfactory (≥ 30 patients included). (1 score)

b) Not justified (< 30 patients included). (0 score)

1. Non-Response rate

a) The response rate is satisfactory (≥95%). (1 Score)

b) The response rate is unsatisfactory (<95%), or no description. (0 Score)

1. Ascertainment of the measurement survey:

a) Validated survey. (2 scores)

b) Non-validated survey, but the survey is available or described. (1 score)

c) No description of the survey. (0 score)

Outcome: (Maximum 3 stars)

1. Assessment of the outcome:

a) Independent blind assessment. (2 scores)

b) Record linkage. (2 scores)

c) Self report. (1 score)

d) No description. (0 score)

1. Statistical test:

a) The statistical test used to analyse the data is clearly described and appropriate. (1 score)

b) The statistical test is not appropriate, not described or incomplete. (0 score)
